# Renal post-mortem findings in myeloproliferative and myelodysplastic/myeloproliferative neoplasms

**DOI:** 10.1007/s00428-021-03129-y

**Published:** 2021-06-23

**Authors:** Fermin Person, Sara C. Meyer, Helmut Hopfer, Thomas Menter

**Affiliations:** 1 Institute of Medical Genetics and Pathology, University Hospital Basel, University of Basel, Schönbeinstrasse 40, 4031 PathologyBasel, Switzerland; 2 Department of Hematology, University Hospital Basel, Basel, Switzerland

**Keywords:** Diffuse glomerulosclerosis, Acute tubular injury, Myeloproliferative neoplasm, Nephropathology

## Abstract

Myeloproliferative neoplasms (MPN) are a heterogeneous group of hematological disorders presenting with an increased proliferation in one or several hematological cell lines. Renal manifestations of MPN have not been fully characterized so far. To morphologically assess the potential renal involvement in MPN patients, we analyzed histomorphological findings of a post-mortem cohort (n = 57) with a disease history of Philadelphia-negative MPN including polycythaemia vera, primary myelofibrosis, essential thrombocythemia, or chronic myelomonocytic leukemia (CMML). Seven (12.2%) patients presented with a pattern of diffuse glomerulosclerosis not attributable to diabetic or hypertensive nephropathy. Weak C4d staining suggestive for chronic thrombotic microangiopathy (TMA) was observed in 4/7 cases. Glomerulonephritis was excluded by light microscopy and immunohistochemistry. Patients with a pattern of diffuse glomerulosclerosis did not differ from the rest of the cohort regarding MPN subtype, disease duration, age, or sex. No significant proteinuria had been observed before death. Further findings attributed to MPNs were extramedullary hematopoiesis (n = 5; 8.8%) and tumor involvement in advanced disease (n = 4; 7.0%). Other common findings included arteriolosclerosis (n = 18; 31.6%) and signs of shock (n = 8; 14.0%). To our knowledge, this study is so far the largest investigating renal findings in MPN patients. There may be a causal relationship between idiopathic diffuse glomerular sclerosis and MPN, although its clinical significance and pathophysiology remain uncertain with TMA probably being relevant in a subgroup of cases. Our findings demonstrate the spectrum of renal findings in MPN from early to terminal disease of which hematologists should be aware of in daily clinical practice.

## Introduction

Myeloproliferative neoplasms (MPN) are clonal hematopoietic stem cell disorders presenting with dysregulated proliferation of one or several lines of myeloid blood cells. Entities include chronic myelogenous leukemia (CML), polycythaemia vera (PV), primary myelofibrosis (PMF), essential thrombocythemia (ET), and the very rare forms of chronic neutrophilic or eosinophilic leukemia [1]. Besides these classical subtypes of MPN, there are entities showing both features of myeloproliferative and myelodysplastic disorders of the bone marrow, which are categorized as myelodysplastic/myeloproliferative neoplasms (MPN/MDS) according to the WHO classification. The most common subtype of this group is chronic myelomonocytic leukemia (CMML) which presents with persistent monocytosis in addition to myelodysplastic features.

The major known clinical complications include risk of thrombosis, extramedullary hematopoiesis, extensive bone marrow fibrosis, and transformation into acute leukemia [2]. Furthermore, CML requires immediate active treatment by BCR-ABL tyrosine kinase inhibitors. Renal involvement has recently received increased awareness as it has been shown to be an independent predictor of inferior outcome [3].

Systematic histopathological examination of kidneys of MPN patients has not been reported for many years with the exception of anecdotal reports or small case series. In 2011, Said et al. published a series of eleven indication biopsies of MPN patients [4]. The patients had been biopsied due to nephrotic range proteinuria (i.e., > 3 g/day) or chronic renal failure. Reported histopathological findings encompass features of mesangial sclerosis with or without accompanying focal segmental sclerosis (FSGS) or thrombotic microangiopathy in the majority of patients as well as the presence of intracapillary hematopoietic cells. The authors concluded that MPN-related glomerulopathy is a late manifestation of the disease pathway seen in fibrosis of the bone marrow in MPN. Recently, a German group investigated a cohort of indication biopsies of 29 MPN and CMML cases [5]. Renal symptoms were similarly comprised proteinuria, microhematuria, and chronic kidney disease. The authors demonstrated a high prevalence of acute or chronic endothelial glomerular damage alongside with mesangial sclerosis. Podocytopathies, either minimal change nephropathy or FSGS with effacement of podocyte foot processes (“primary FSGS”), was seen in a third of the patients. Interestingly, a fifth of the patients presented with either IgA-nephropathy or infection-related glomerulonephritis.

In contrast to these studies on indication biopsies, a systematic overview on renal histopathology of MPN and MPN/MDS patients is still lacking and the prevalence of renal complications in this patient group has not been fully elucidated to date.

In order to address this issue, we provide data on an unselected cohort of MPN and MPN/MDS patients (regardless of clinical manifestation of kidney disease). We retrospectively analyzed renal histopathology in a cohort of 57 autopsies of patients with a diagnosis of MPN (excluding cases of CML) or MPN/MDS.

## Material and methods

### Patient cohort

We reviewed all autopsy reports performed at our institution between 2000 and 2020 in order to identify patients with a diagnosis of MPN (PV, ET, PMF, MPN-U) or MDS/MPN, diagnosed either at lifetime or at the time of autopsy (n = 57). Altogether, 57 patients were included in the present study. Renal function estimation was calculated using the MDRD formula (Modification of Diet in Renal Disease [6]) considering available data of the patients. Kidney function was then grouped according to KDIGO guidelines [7]. Clinical details are shown in Table 1. Clinical data concerning comorbidities, renal function, and duration of disease were gathered from previous pathology reports and electronically available patient files.

**Table 1:** General clinical characteristics and autopsy findings of our cohort

Findings	
Male/female	32/25
Age	Mean: 74, median: 77, range: 33–96 years
Duration of disease	Mean: 7 years, median: 3 years, range: < 1–50 years
Type of disease	CMML: 18
	ET: 1
	PV: 16
	PMF: 16
	MPN other: 6
Hematopoetic cell transplantation	11/57
Cause of death	Cardiac disease: 11/57
	HCT-related: 2/57
	Infectious diseases: 27/57
	Hemorrhagic shock: 4/57
	Lung embolism: 9/57
	Tumor progression: 4/57
Kidney function*	G1: 17/55
	G2: 27/55
	G3a: 6/55
	G3b: 3/55
	G4: 2/55
Idiopathic diffuse glomeruloclerosis	7/57
Diabetic glomerulosclerosis	2/57
Diabetes	2/57
Arteriolosclerosis	18/57
Shock signs	8/57
Florid pyelonephritis	2/57
Extramedullary hematopoesis in the kidney	5/57
Infiltration of the kidney by blastoid cells	3/57
Other non-specific findings**	29/57
No renal involvement	9/57

*Laboratory parameters were available of 55/57 patients.

**This includes simple cysts, small vascular scars, mild nephrocalcinosis, and papillary adenoma.

### Morphological evaluation

All cases were examined using routine histochemical stains (hematoxylin–eosin (HE), periodic acid-Schiff (PAS), and trichrome stains). Cases in which the diagnosis of diffuse mesangial sclerosis was made were additionally stained by methenamine silver stain and immunohistochemistry for IgA (A0262, Agilent, Santa Clara, CA, USA), IgG (A0423, Agilent), C3 (A0062, Agilent), and C5b-9 (AE11, Quidel, San Diego, CA, USA) to exclude glomerulonephritis. Cases with diffuse mesangial glomerulosclerosis as well as cases showing clear features of thrombotic microangiopathy (TMA) were stained for C4d (BI-RC4D, Biomedica, Vienna, Austria). Morphologic features were only evaluated in areas without autolytic changes. Completely autolytic cases were excluded from the study.

### Statistical evaluation

Data analysis was performed using the R and R Studio software (expss and dplyr packages). Mean, median, Welch two sample t test, and Pearson’s Chi-squared test with Yates’ continuity correction were calculated. Results with p < 0.05 were considered statistically significant.

## Results

### Clinical characteristics

Our cohort consisted of 32 (56%) male and 25 (44%) female patients with a mean age of 74 years (33–96 years, median 77 years). Diagnoses included 18 (32%) patients with CMML, 16 (28%) patients each with PV and PMF, one patient with ET (2%), and in 6 (10%) patients with MDS/MPN unclassifiable. The mean onset of disease was 7 years prior to death (median 3 years). Eleven patients had been treated with hematopoietic cell transplantation. The main cause of death was infectious disease (27/57, 47%) and cardiac diseases (11/57, 19%). Of the deaths resulting from infectious disease, bacterial pneumonia was the most frequent cause of death (19 cases, 70% of all infection related deaths), followed by fungal pneumonia (5 cases, 18%). One case each of CMV-related pneumonia (4%), endocarditis (4%), and concurrent meningitis and pyelonephritis (4%) was also evident. Other causes of death were HCT-related (2/57, 4%), haemorrhagic shock (4/57, 7%), lung embolism (9/57, 16%), and tumor progression (4/57, 7%). A good renal function (not considering acute renal failure prior to death) was documented in 17 of 55 patients with available laboratory data. Mild or moderate renal impairment was present in 27 and 9 patients, respectively. Two patients had known severe renal insufficiency.

### Presence of diffuse mesangial glomerulosclerosis

**Fig. 1:**
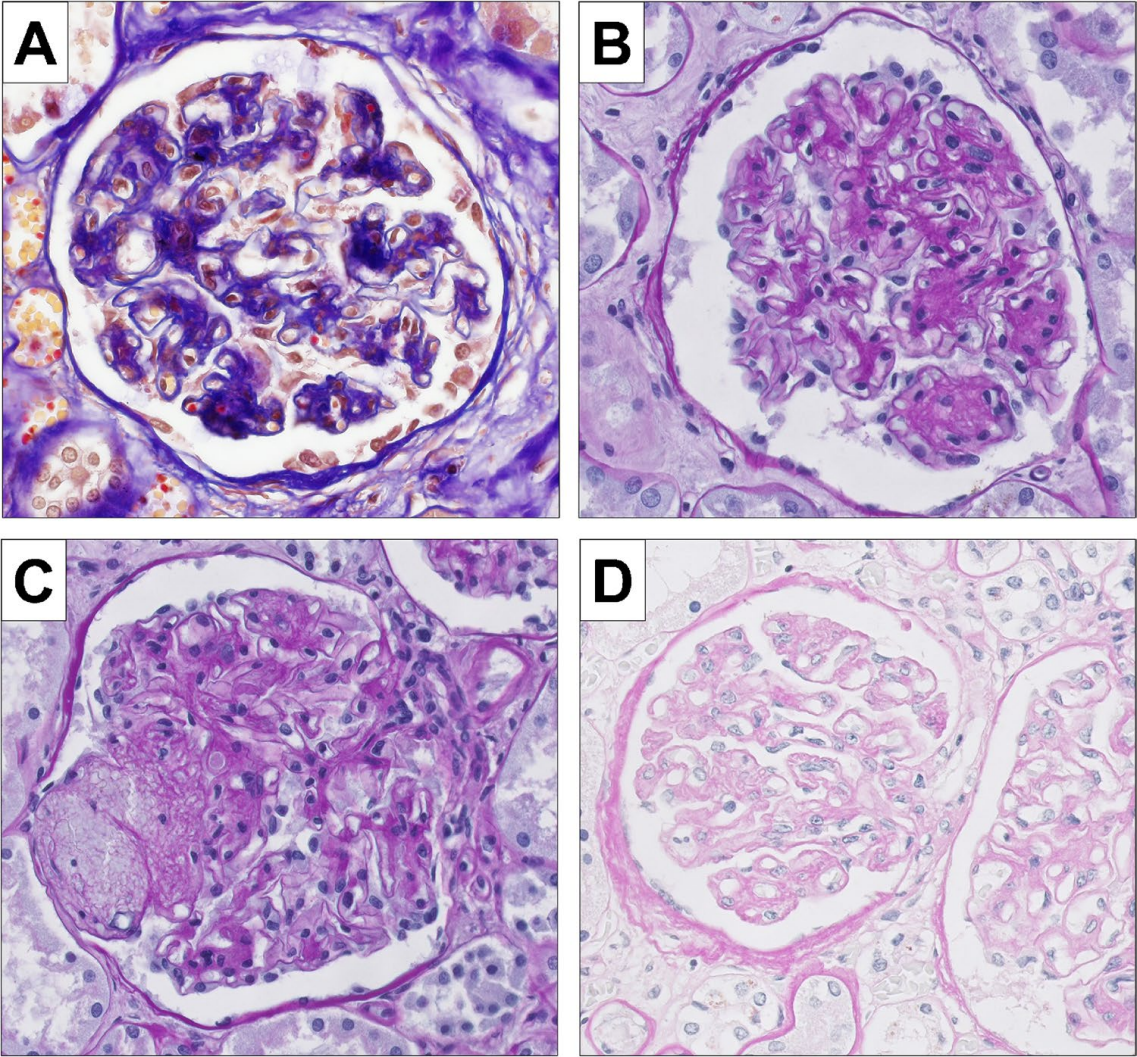
Features of diffuse mesangial sclerosis. **A** and **B** Mesangial matrix expansion can be seen in the trichrome (**A**) and the PAS stain (**B**) (× 400); **C** another case of diffuse mesangial sclerosis showing mesangiolysis (arrow) hinting at chronic endothelial damage [11] (PAS, × 400); **D** prominent wrinkling of glomerular capillary basement membranes in addition to mild mesangial matrix expansion also pointing at chronic TMA (PAS, × 400)

**Fig. 2:**
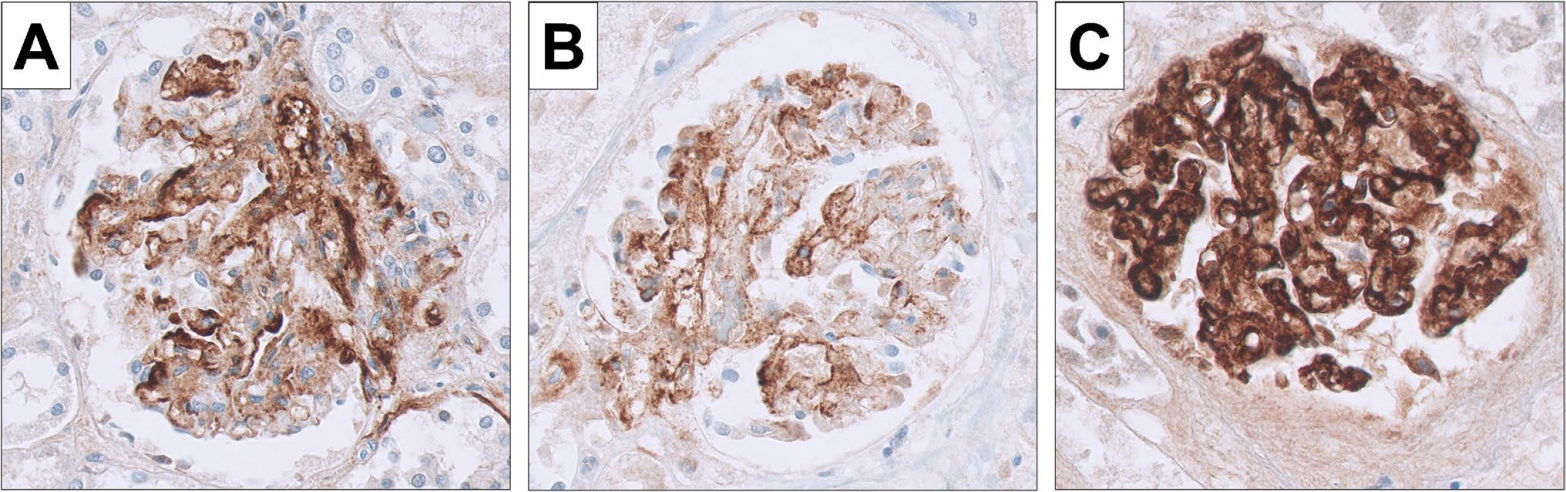
Staining for C4d. **A** and **B** Cases of diffuse mesangial sclerosis showing segmental and focal staining for C4d both in the mesangium and the capillary walls (× 400); **C** control case for C4d staining of overt TMA showing strong staining for C4d in the capillary walls (× 400)

Diffuse mesangial glomerulosclerosis was observed in 7/57 (12%) patients (Fig. 1A and B). In all of these patients, there was neither morphological nor clinical evidence of diabetes mellitus or severe hypertensive disease. Immune-complex-associated mesangioproliferative or membranous glomerulonephritis was ruled out by immunohistochemistry. There was no statistically significant difference regarding the underlying disease which was evenly distributed among PV, PMF, and CMML. According to available clinical information, there was no past medical history of proteinuria or microhematuria in these patients. No patient had a history of monoclonal gammopathy of unknown significance (MGUS) or overt plasma cell myeloma thus also providing no evidence for light chain nephropathy. They also did not show clinical features of thrombotic microangiopathy (TMA). One case showed extensive older mesangiolysis (Fig. 1C); three cases showed mildly thickened and wrinkled basement membranes (Fig. 1D) which might be attributable to chronic TMA. Staining for C4d of these cases showed weak segmental peripheral and mesangial staining for C4d compared to strong C4d staining of the case of overt TMA (Fig. 2).

### Other renal findings related to the underlying hematologic disease

**Fig. 3:**
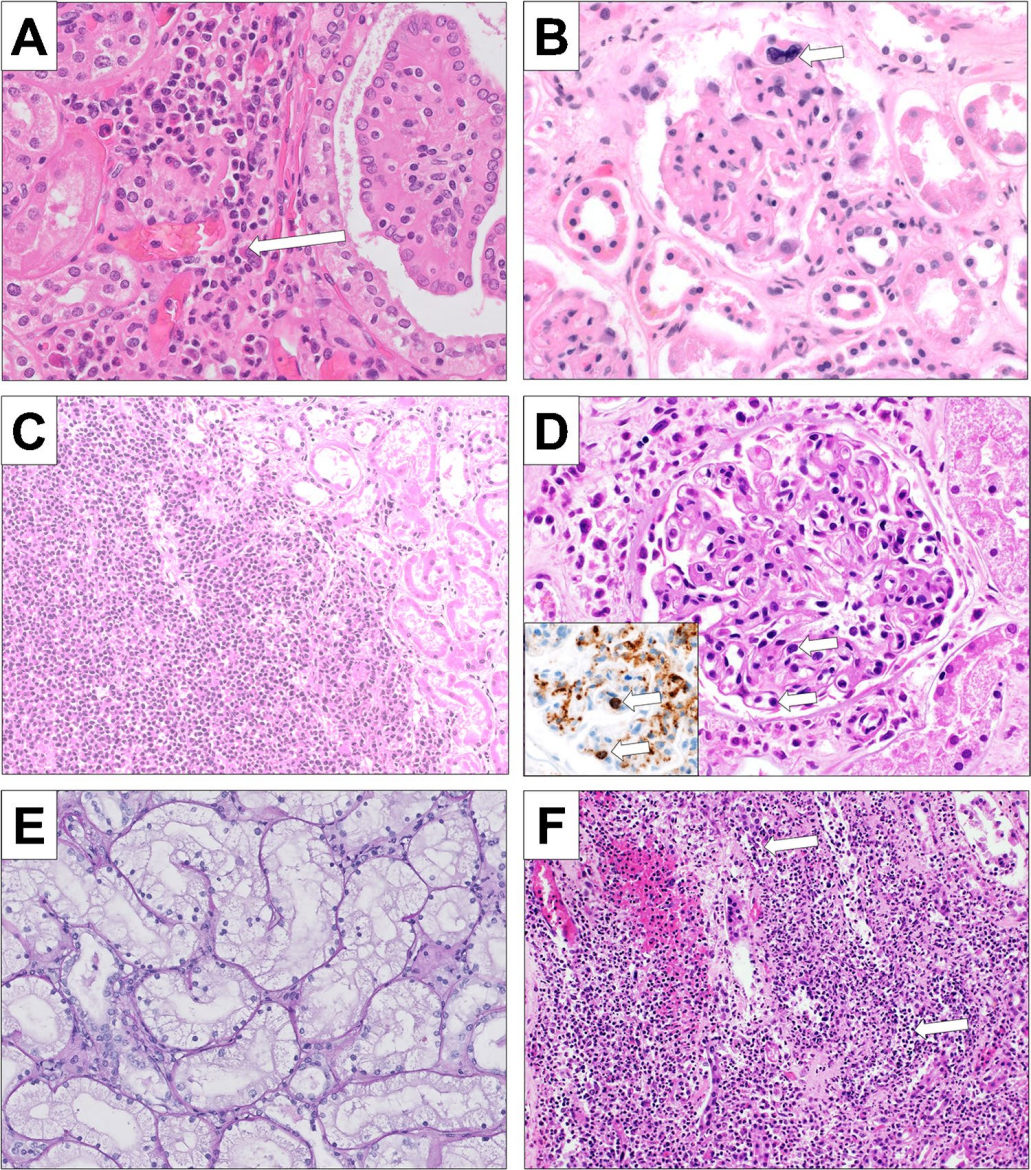
Other morphologic findings in our cohort. **A** Focus of extramedullary hematopoiesis showing myeloid and erythroid cells as well as a megakaryocyte (arrow) (H&E, × 400). **B** Extramedullary hematopoiesis showing a megakaryocyte (arrow) in a capillary loop of the glomerulus (H&E, × 400); **C** Kidney infiltrates of blasts in a patient with transformation of PMF into AML (H&E, × 200); **D** Glomerulus showing intracapillary blasts and myeloid precursors (arrow) of a case with transformation into AML (H&E, × 400); the insert confirms the blasts in a CD34 immunohistochemical stain (arrow, × 400); **E** severe acute tubular damage of the osmotic nephrosis type showing fine-vaculoated cytoplasm of the tubuli (H&E, × 200); **F** acute pyelonephritis showing a dense infiltrate of neutrophils destroying the basement membrane of tubuli (arrow) (H&E, × 200)

Five patients (9%) showed histological evidence of extramedullary hematopoiesis (Fig. 3A) which was located in the renal cortex. In three patients (7%), there was an infiltration of malignant cells in the kidney, primarily presenting as transformed acute leukemia (Fig. 3B). These were focal infiltrates occupying < 5% of the renal parenchyma. They partially showed a destructive growth pattern infiltrating primarily the tubules. In few cases, circulating hematopoietic cells such as megakaryocytes (Fig. 3C) and blastoid cells (Fig. 3D) were observed in glomerular capillaries. One case showed focal infiltrates of chronic lymphocytic leukemia (CLL). In one case, there were renal infiltrates of CMML.

### Distribution of other findings

The most common non-specific finding was arteriolosclerosis. Four (7%) patients had a clinical history of diabetes mellitus; two patients (4%) showed nodular diabetic glomerulosclerosis. Eight patients (14%) displayed renal signs of shock (Fig. 3E). Other findings included acute pyelonephritis (n = 2, 4%) (Fig. 3F) and unilocular simple cysts.

### Pathologies in patients having received hematologic cell transplantation

Patients who received with HCT were significantly younger (mean no HCT 77 years, HCT 62, p = 0.001) and showed a trend towards a higher incidence of shock signs. Of the 11 patients treated with HCT, 2 showed signs of diffuse glomerulosclerosis (2/11, 18%) (Table 2). A detailed summary of the findings in HCT treated patients is provided in Table 3.

**Table 2:** Characteristics of patient diagnosed at autopsy with idiopathic diffuse glomerular sclerosis

	No diffuse glomerulosclerosis	Diffuse glomerulosclerosis	Test for significance
Male/Female	3/4	29/21	X-squared = 0.12219, p value = 0.7267
Age	Mean: 73.5, median: 76.5, range: 33–96 years	Mean: 79, median: 83, range: < 1–28 years	p value = 0.1151
Duration of disease	Mean: 7.2, median: 2, range: < 1–50 years	Mean: 9, median: 5, range: 70–86 years	p value = 0.6547
Type of disease	CMML: 16/50 ET: 1/50 PV: 14/50 PMF: 13/50 MPN other: 6/50	CMML: 2/7 ET: 0/7 PV: 2/7 PMF: 3/7 MPN other: 0/7	X-squared = 8.1103, p value = 0.7034
Hematopoetic cell transplantation	9/50	2/7	X-squared = 0.023254, p value = 0.8788
Cause of death	Cardiac disease: 10/50 HCT-related: 2/50 Infectious diseases: 25/50 Hemorrhagic shock: 3/50 Lung embolism: 7/50 Tumor progression: 3/50	Cardiac disease: 1/7 HCT-related: 0/7 Infectious diseases: 2/7 Hemorrhagic shock: 1/7 Lung embolism: 2/7 Tumor progression: 1/7	X-squared = 3.0063, p value = 0.699
Kidney function	G1: 15/48 G2: 25/48 G3a: 3/48 G3b: 3/48 G4: 2/48	G1: 2/7 G2: 2/7 G3a: 3/7 G3b: 0/7 G4: 0/7	
Diabetic glomerulosclerosis	2/50	0/7	X-squared = 7.8287e-32, p value = 1
Diabetes	2/50	0/7	X-squared = 7.8287e-32, p value = 1
Arteriolosclerosis	15/50	3/7	X-squared = 0.063159, p value = 0.8016
Shock signs	7/50	1/7	X-squared = 2.1909e-31, p value = 1
Acute pyelonephritis	2/50	0/7	X-squared = 7.8287e-32, p value = 1
Extramedullary hematopoesis in the kidney	5/50	0/7	X-squared = 0.026464, p value = 0.8708
Infiltration of the kidney by blastoid cells	3/50	0/7	X-squared = 1.5657e-31, p value = 1
Other non-specific findings	29/50	0/7	X-squared = 6.1072, p value = 0.01346
No renal involvement	9/50	0/7	X-squared = 0.44871, p value = 0.503

**Table 3:** Characteristics of patients with and without stem cell transplantation

	No hematopoetic cell transplantation	Hematopoetic cell transplantation	Test for significance
Male/Female	23/23	9/2	X-squared = 2.4721, p value = 0.1159
Age	Mean: 77, median: 78, range 54–96 years	Mean: 62, median: 66, range: 33–72 years	p value = 0.001097
Duration of disease	Mean: 8, median: 3, range: < 1–50 years	Mean: 6, median: 2, range: < 1–17 years	p value = 0.4739
Type of disease	CMML: 12/46 ET: 1/46 PV: 14/46 PMF: 13/46 MPN other: 6/46	CMML: 6/11 ET: 0/11 PV: 2/11 PMF: 2/11 MPN other: 1/11	X-squared = 10.983, p value = 0.4447
Idiopathic diffuse glomerulosclerosis	5/46	2/11	X-squared = 0.023254, p value = 0.8788
Cause of death	Cardiac disease: 10/46 HCT-related: 2/46 Infectious diseases: 20/46 Hemorrhagic shock: 3/46 Lung embolism: 9/46 Tumor progression: 4/46	Cardiac disease: 1/11 HCT-related: 2/11 Infectious diseases: 7/11 Hemorrhagic shock: 1/11 Lung embolism: 0/11 Tumor progression: 0/11	X-squared = 13.053, p value = 0.02288
Kidney function	G1: 12/44 G2: 22/44 G3a: 5/44 G3b: 3/44 G4: 2/44	G1: 5/11 G2: 5/11 G3a: 1/11 G3b: 0/11 G4: 0/11	
Diabetic glomerulosclerosis	2/46	0/11	X-squared = 3.492e-29, p value = 1
Diabetes	2/46	0/11	X-squared = 3.492e-29, p value = 1
Arteriolosclerosis	17/46	1/11	X-squared = 2.0309, p value = 0.1541
Shock signs	4/46	4/11	X-squared = 3.5726, p value = 0.05874
Acute pyelonephritis	2/46	0/11	X-squared = 3.492e-29, p value = 1
Extramedullary hematopoesis in the kidney	5/46	0/11	X-squared = 0.30426, p value = 0.5812
Infiltration of the kidney by blastoid cells	3/46	0/11	X-squared = 0.12766, p value = 0.7209
Other non-specific findings	25/46	4/11	X-squared = 0.54191, p value = 0.4616
No renal involvement	7/46	2/11	X-squared = 5.3534e-32, p value = 1

## Discussion

This post-mortem study provides an overview of renal findings of MPN and MDS/MPN patients. In contrast to indication biopsies, this cohort represents an unbiased cohort not selected for clinically relevant renal impairment. Kidney function was good or only showed mild impairment in the majority of patients with a glomerular filtration rate (GFR) of more than 60 ml/min in 44/55 evaluable patients.

Previous evidence suggests that diffuse mesangial sclerosis and focal segmental glomerulosclerosis are occurring in MPN patients. The pathogenesis of MPN-related glomerulopathy is still a matter of debate. It is currently surmised that it may develop as a result of chronic endothelial damage and a combination of fibrosis-inducing factors [8]. The largest recently published study on indication biopsies of patients manifesting with significant proteinuria, hematuria, or impaired kidney function reports a high rate of both acute and chronic TMA-related changes with at least a quarter of cases presenting with renal findings attributed to TMA [5].

Another feature encountered in MPN patients is extramedullary hematopoiesis of the kidney. An earlier autopsy study reported a frequency of around one-third of patients [9]. However, in one series of 14 indication biopsies, it was very rare [10]. The authors described findings of acute and chronic TMA, focal segmental sclerosis, and two cases of fibrillary-like glomerulonephritis. Whether extramedullary hematopoiesis itself has a direct detrimental effect on the kidney is not clear yet. The abovementioned series reported one case of mass-forming extramedullary hematopoiesis which might affect kidney function by direct compression of tissue as did perirenal extramedullary hematopoiesis involving the pelvic/ureteral area causing obstruction [9]. In our series, extramedullary hematopoiesis was focal and not mass forming, it was observed both in the renal parenchyma and the perirenal adipose tissue. Additionally, we observed extensive infiltrates of blasts in cases transforming to overt acute leukemia in three cases.

In our cohort, there were no clinically or histopathological suggestive signs of florid TMA. We describe one case with mesangiolysis which can represent the outcome of ongoing endothelial damage [11], furthermore capillary wrinkling was observed which may also be a consequence of TMA-episodes in the past. As electron microscopy could not be performed in this autopsy-based-study, we performed staining for C4d which has been described to be positive in cases of TMA [12]. Although expression of C4d was less prominent in comparison to a control case of overt TMA, the focal staining in cases of diffuse mesangial sclerosis showing mesangiolysis or capillary wrinkling might strengthen the hypothesis that chronic endothelial damage related to TMA contributes to the development of diffuse mesangial sclerosis in some patients.

Thus, a causal relationship between diffuse mesangial sclerosis and MPNS/CMML is possible, although it may not become clinically evident due to renal impairment or significant proteinuria, in most cases considering the relatively high rate of this finding in our cohort and the overall scarcity of renal biopsies of MPN/CMML patients.

In concordance with our previous study on the post-mortem findings of HCT patients [13], we confirm a higher rate of acute tubular damage in MPN-patients treated with HCT. These patients had prominent features of fulminant graft versus host disease (GvHD) or tumor relapse which may be a possible explanation for the acute kidney injury seen. As we could also see features of diffuse mesangial glomerulosclerosis in HCT patient without persistent MPN or MPN/MDS, it can be concluded that diffuse mesangial glomerulosclerosis is a chronic and persistent finding even years after HCT.

In conclusion, this study provides an overview of renal histomorphologic findings in MPN and CMML patients. Although diffuse glomerulosclerosis was observed in > 10% of patients, it did not result in evident proteinuria, implying that its clinical impact might be limited. Furthermore, we describe features of chronic TMA in a subset of patients. Oncologists should keep in mind infectious complications and direct tumor involvement, especially in terminal patients.

## Data Availability

The datasets used and/or analyzed during the present study are available from the corresponding author on reasonable request.

## References

[1] Spivak JL (2017). Myeloproliferative Neoplasms. N Engl J Med.

[2] Tefferi A, Barbui T (2019). Polycythemia vera and essential thrombocythemia: 2019 update on diagnosis, risk-stratification and management. Am J Hematol.

[3] Lucijanic M, Galusic D, Krecak I, Sedinic M, Holik H, Perisa V, et al. Reduced renal function strongly affects survival and thrombosis in patients with myelofibrosis. Ann Hematol.10.1007/s00277-020-04239-4 10.1007/s00277-020-04239-4 32862283

[4] Said SM, Leung N, Sethi S, Cornell LD, Fidler ME, Grande JP (2011). Myeloproliferative neoplasms cause glomerulopathy. Kidney Int.

[5] Buttner-Herold M, Sticht C, Wiech T, Porubsky S. Renal disease associated with myeloproliferative and myelodysplastic/myeloproliferative neoplasia. Histopathology.10.1111/his.14282 10.1111/his.14282 33078472

[6] Levey AS, Coresh J, Greene T, Stevens LA, Zhang YL, Hendriksen S (2006). Using standardized serum creatinine values in the modification of diet in renal disease study equation for estimating glomerular filtration rate. Ann Intern Med.

[7] Stevens PE, Levin A (2013). Kidney Disease: Improving Global Outcomes Chronic Kidney Disease Guideline Development Work Group M. Evaluation and management of chronic kidney disease: synopsis of the kidney disease: improving global outcomes 2012 clinical practice guideline. Ann Intern Med.

[8] Koschmieder S, Chatain N (2020). Role of inflammation in the biology of myeloproliferative neoplasms. Blood Rev.

[9] Pitcock JA, Reinhard EH, Justus BW, Mendelsohn RS (1962). A clinical and pathological study of seventy cases of myelofibrosis. Ann Intern Med.

[10] Alexander MP, Nasr SH, Kurtin PJ, Casey ET, Hernandez LP, Fidler ME (2015). Renal extramedullary hematopoiesis: interstitial and glomerular pathology. Mod Pathol.

[11] Morita T, Yamamoto T, Churg J (1998). Mesangiolysis: an update. Am J Kidney Dis.

[12] Drachenberg CB, Papadimitriou JC, Chandra P, Haririan A, Mendley S, Weir MR (2019). Epidemiology and pathophysiology of glomerular C4d staining in native kidney biopsies. Kidney Int Rep.

[13] Girsberger M, Halter JP, Hopfer H, Dickenmann M, Menter T (2018). Kidney pathology after hematologic cell transplantation-a single-center observation study of indication biopsies and autopsies. Biol Blood Marrow Transplant.

